# Pooled-DNA sequencing identifies novel causative variants in *PSEN1*, *GRN *and *MAPT *in a clinical early-onset and familial Alzheimer's disease Ibero-American cohort

**DOI:** 10.1186/alzrt137

**Published:** 2012-08-20

**Authors:** Sheng Chih Jin, Pau Pastor, Breanna Cooper, Sebastian Cervantes, Bruno A Benitez, Cristina Razquin, Alison Goate, Ibero-American Alzheimer Disease Genetics Group Researchers, Carlos Cruchaga

**Affiliations:** 1 Department of Psychiatry, Washington University School of Medicine, 660 South Euclid Avenue B8134, St. Louis, MO 63110, USA; 2 Neurogenetics Laboratory, Division of Neurosciences, Center for Applied Medical Research, University of Navarra, Campus Universitario, 31080 Pamplona, Navarra, Spain; 3 Department of Neurology, Clínica Universidad de Navarra, University of Navarra School of Medicine, Campus Universitario, 31080 Pamplona, Navarra, Spain; 4 CIBERNED, Centro de Investigación Biomédica en Red de Enfermedades Neurodegenerativas, Instituto de Salud Carlos III, C/Sinesio Delgado, 4, 28029, Madrid, Spain

## Abstract

**Introduction:**

Some familial Alzheimer's disease (AD) cases are caused by rare and highly-penetrant mutations in *APP*, *PSEN1*, and *PSEN2*. Mutations in *GRN *and *MAPT*, two genes associated with frontotemporal dementia (FTD), have been found in clinically diagnosed AD cases. Due to the dramatic developments in next-generation sequencing (NGS), high-throughput sequencing of targeted genomic regions of the human genome in many individuals in a single run is now cheap and feasible. Recent findings favor the *rare variant-common disease *hypothesis by which the combination effects of rare variants could explain a large proportion of the heritability. We utilized NGS to identify rare and pathogenic variants in *APP*, *PSEN1*, *PSEN2*, *GRN*, and *MAPT *in an Ibero-American cohort.

**Methods:**

We performed pooled-DNA sequencing of each exon and flanking sequences in *APP, PSEN1, PSEN2, MAPT *and *GRN *in 167 clinical and 5 autopsy-confirmed AD cases (15 familial early-onset, 136 sporadic early-onset and 16 familial late-onset) from Spain and Uruguay using NGS. Follow-up genotyping was used to validate variants. After genotyping additional controls, we performed segregation and functional analyses to determine the pathogenicity of validated variants.

**Results:**

We identified a novel G to T transition (g.38816G>T) in exon 6 of *PSEN1 *in a sporadic early-onset AD case, resulting in a previously described pathogenic p.L173F mutation. A pathogenic p.L392V mutation in exon 11 was found in one familial early-onset AD case. We also identified a novel CC insertion (g.10974_10975insCC) in exon 8 of *GRN*, which introduced a premature stop codon, resulting in nonsense-mediated mRNA decay. This *GRN *mutation was associated with lower GRN plasma levels, as previously reported for other *GRN *pathogenic mutations. We found two variants in *MAPT *(p.A152T, p.S318L) present only in three AD cases but not controls, suggesting that these variants could be risk factors for the disease.

**Conclusions:**

We found pathogenic mutations in *PSEN1*, *GRN *and *MAPT *in 2.33% of the screened cases. This study suggests that pathogenic mutations or risk variants in *MAPT *and in *GRN *are as frequent in clinical AD cases as mutations in *APP*, *PSEN1 *and *PSEN2*, highlighting that pleiotropy of *MAPT *or *GRN *mutations can influence both FTD and AD phenotypic traits.

## Introduction

Alzheimer's disease (AD) is the most common form of dementia, affecting more than 13% of individuals age 65 years or older and 30% to 50% of individuals age 80 or older [1,2]. The number of affected individuals is estimated to double by 2025; thus, AD is rapidly becoming a serious threat to health care in developed countries [2]. Since the number of patients and health-care costs are projected to increase significantly, effective therapies are urgently needed. Understanding how genetic risk factors affect the disease process will help to identify novel targets for therapies, to elucidate the nature of aging, and to extend the healthy active life span.

AD is often classified on the basis of the age at onset (AAO); early-onset AD (EOAD) is defined as AAO of not more than 65 years, and late-onset AD (LOAD) is defined as AAO of more than 65 years. Even though 99% of AD cases are late-onset, studies of rare autosomal dominant familial EOAD have provided valuable insights into the pathogenesis of AD. Mutations in amyloid-β precursor protein (*APP*), presenilin 1 (*PSEN1*), and presenilin 2 *(PSEN2*) were initially discovered in familial EOAD, although additional studies have also identified pathogenic mutations in these genes in late-onset families in addition to the APOE4 allele [3-9]. Progranulin (*GRN*) and microtubule-associated protein tau (*MAPT*) mutations are associated with familial frontotemporal dementia, but recently some have also been found in clinically diagnosed AD cases [10,11], and a recent study suggested that mutations in *MAPT *and *GRN *can be found in clinical AD with a frequency comparable to that of mutations in *APP*, *PSEN1*, and *PSEN2 *[7].

**Figure 1:**
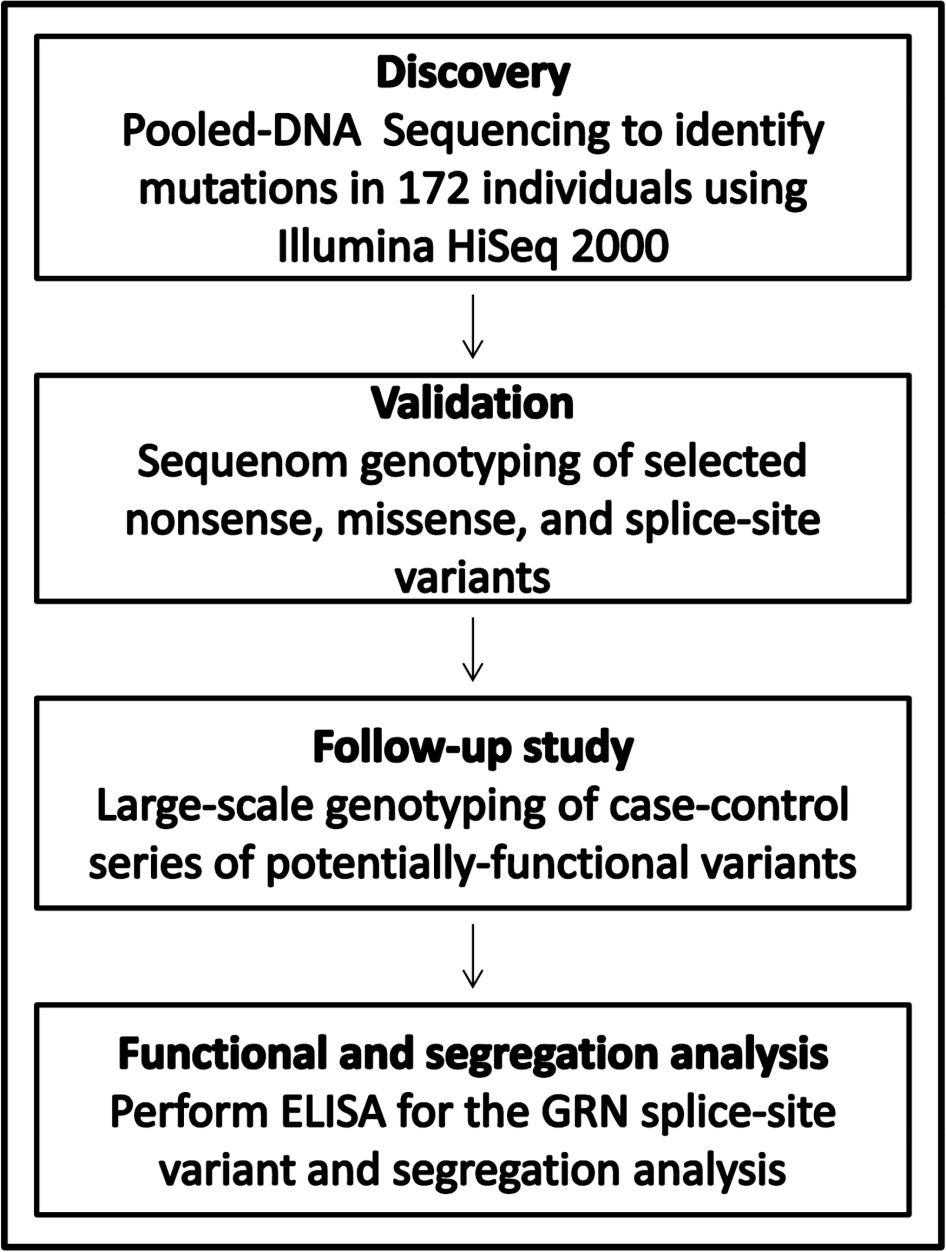
**Schematic of the study design**. Pooled-DNA sequencing was performed in a single DNA pool of 172 individuals to identify known pathogenic or novel functional variants by using Illumina HiSeq 2000. The SPLINTER software was used to call the variants. High-confident variants were selected for Sequenom genotyping. For those validated functional variants, follow-up genotyping was performed in large case-control series to infer and compare the frequencies. Segregation analysis was then performed to determine whether disease status segregates with risk alleles. Enzyme-linked immunosorbent assays (ELISAs) were used to evaluate the impact of novel *GRN *splice-site mutation on the changes of *GRN *plasma levels. *GRN*, progranulin; SPLINTER, short indel prediction by large deviation inference and nonlinear true frequency estimation by recursion.

In this study, we aimed to examine the frequency of causative mutations in autosomal dominant dementia genes in independent clinical series from Spain and Uruguay. We performed pooled-DNA sequencing in 172 AD cases in *APP*, *PSEN1*, *PSEN2*, *GRN*, and *MAPT *in order to identify known pathogenic mutations and potentially functional novel variants associated with disease risk. For those validated variants, follow-up genotyping was conducted in additional AD cases and non-demented older controls with Spanish ancestry to infer and compare mutation frequencies. We also genotyped available relatives of the mutation carriers to determine whether these mutations segregate with the diagnosis of AD (Figure 1). Finally, enzyme-linked immunosorbent assay (ELISA) was performed to test the association between the status of the novel *GRN *mutation and *GRN *plasma levels.

## Materials and methods

### Sample selection

DNA was collected from subjects of Spanish descent who were recruited between 2000 and 2010 from the departments of neurology of the collaborating centers from Spain (n = 161) and Uruguay (n = 11). Samples were selected from individuals with neuropathologically confirmed AD or from individuals with the earliest AAO along with a diagnosis of probable AD within a family. The cohort included 15 (8.98%) familial EOAD, 136 (81.44%) sporadic EOAD, and 16 (9.58%) familial LOAD cases and five others who were autopsy-confirmed AD cases (Table 1). Diagnosis of probable AD was made in accordance with standard clinical research criteria [12]. All individuals gave their written informed consent for participating in the study, which was approved by the institutional review board of Washington University in St. Louis.

**Table 1:** Demographic characteristics of the cohort of 172 sequenced samples

**Demographic characteristics**		
Age at onset of AD	Years	
Mean	59.56	
Standard deviation	8.19	
Range	29.5-87.5	
		
	Number	Percentage
Diagnosis		
Familial early-onset AD (onset ≤65 years)	15	8.98%
Sporadic early-onset AD (onset ≤65 years)	136	81.44%
Familial late-onset AD (onset >65 years)	16	9.58%
		
Clinical AD	167	97.09%
Autopsy-confirmed	5	2.91%
APOE		
ε2ε2+ε2ε3	3	1.75%
ε3ε3	79	45.93%
ε3ε4	66	38.37%
ε4ε4	24	13.95%
Birth country		
Spain	161	93.60%
Uruguay	11	6.40%
Gender		
Male	62	36.05%
Female	105	61.05%
Unknown	5	2.91%

AD, Alzheimer's disease; APOE, apolipoprotein E.

### Pooled sequencing

Pooled-DNA sequencing was performed as previously described [7,13]. The concentration of genomic DNA was quantified by Quant-iT™ PicoGreen (Invitrogen Corporation, Carlsbad, CA, USA) reagent, and normalized amounts of individual DNA samples were pooled. A single DNA pool with 172 individuals was used as a template for polymerase chain reaction (PCR) amplification of coding regions of genes *APP*, *PSEN1*, *PSEN2*, *MAPT*, and *GRN*. PRIMER3 software was used to design the primers for amplification of each exon. To ensure complete coverage of each exon, primers were at least 50 base pairs (bp) from the intron-exon boundary. Pfu (Agilent Technologies, Santa Clara, CA, USA) enzyme was used for all PCRs to reduce the likelihood of PCR-induced sequence variants. After PCR amplification of selected genomic regions, each PCR product was purified by using QIAquick PCR (Qiagen Inc., Valencia, CA, USA) purification kits and quantified by using Quant-iT™ PicoGreen reagent. The PCR products were combined into one pool in equimolar amounts and ligated by using T4 ligase and T4 Polynucleotide Kinase [14]. At this stage, negative control and positive control amplicons were also added to the pool to generate the error model and to construct the optimal significant cutoff, respectively. Ligated concatemers were randomly fragmented by sonication and prepared for Illumina sequencing on an Illumina HiSeq2000 (Illumina, Inc., San Diego, CA, USA) in accordance with the protocol of the manufacturer. The fold coverage necessary to achieve the optimal positive predictive value for the SNP-calling algorithm was calculated, and a sequencing coverage of 30X per haploid genome was targeted in this study, consistent with the previous finding [14]. Two lanes of Illumina HiSeq2000 sequencing were run to obtain a minimum of 30-fold coverage per allele.

### Sequencing analysis

Sequencing reads (42-bp reads) were mapped back to the reference sequence (GRCh37/hg19) allowing up to three mismatches by using an alignment algorithm developed by Vallania and colleagues [14]. To determine the sensitivity and specificity of this method, positive and negative control vectors were included as PCR products in the pooled-DNA sequencing protocol described above. The negative control reads were used to build up the error model used in the variant calling step, whereas the positive control reads were used to calculate the significant cutoff for optimizing specificity and sensitivity of the analysis. The SPLINTER (short indel prediction by large deviation inference and nonlinear true frequency estimation by recursion) program was used to predict and quantify short insertions, deletions, and substitutions present in the pool. The segregated variants were called by comparing the observed frequency vector to the expected frequency vector calculated by the error model. The maximum likelihood method was used to estimate variant frequencies in the pool samples. SIFT2 was used to predict the effect of variants on protein structure and function, and the Alzheimer Disease & Frontotemporal Dementia (AD&FTD) mutation database [15] was used to annotate the known pathogenic variants. The Exome Variant Server (EVS) [16], SeattleSeq Annotation [17], and 1000 Genomes Project were finally used to exclude known variants. Novel and potentially functional variants were selected for direct genotyping by using Sequenom iPLEX (Sequenom, San Diego, CA, USA).

### Genotyping and segregation analysis

All rare (minor allele frequency of less than 5%) missense, splice-site, and previously identified pathogenic variants called in the pooled-DNA sequencing were genotyped by using Sequenom iPLEX in accordance with standard protocols. We genotyped validated variants in all available family members to determine whether these variants segregate with disease status. Common and synonymous variants were not followed up.

### GRN plasma level measurement

*GRN *plasma levels were measured in duplicate by using an ELISA kit (Human Progranulin ELISA Kit; AdipoGen Inc., Seoul, Korea).

## Results

To identify known pathogenic mutations and novel rare variants, pooled-DNA sequencing was performed for the coding exons and their corresponding flanking regions for *APP*, *PSEN1*, *PSEN2*, *MAPT*, and *GRN *in a total of 15 (8.98%) familial EOAD, 136 (81.44%) sporadic EOAD, 16 (9.58%) familial LOAD, and five autopsy-confirmed AD cases (Table 1). For familial cases, a single index case per family was sequenced. Seventy exons covering 45.3 kb of the target region of *APP*, *PSEN1*, *PSEN2*, *GRN*, and *MAPT *were PCR-amplified by using specific primers and then pooled in equimolar amounts. The pooled amplicons were concatenated and sheared to construct libraries and then sequenced on Illumina HiSeq2000 by using two lanes.

We used the SPLINTER software to perform alignment and call rare variants in the pooled samples [14]. Sequencing reads were mapped back to the reference genome (GRCh37/hg19) by gapped alignments allowing up to 3-bp mismatches by using the SPLINTER aligner. The Illumina sequencing produced 181,854,451 reads in two lanes, and the aligner was able to map 155,431,194 reads (85.5%) back to the reference, resulting in an average 149.8-fold coverage per amplicon (corresponding to 143.5-fold coverage per allele).

Since our goal was to identify novel or functional rare variants, 12 missense, splice-site, or previously confirmed pathogenic variants were selected for direct genotyping by using Sequenom iPLEX (Table 2). These 12 variants were found in 34 out of 172 (19.77%) AD cases and 12 out of 139 (8.63%) non-demented older controls of Spanish descent (Table 2).

**Table 2:** Frequencies of validated variants in the pooled sequencing and follow-up case control series of Spanish descent

Gene-Exon	AA change	rs ID	Codonchange	AD&FTD^a^	Alzheimer's disease cases			Controls
					
					Count (%)	AAO range, years	Diagnoses^b^	Count (%)
*GRN*-8	Ile256IlefsX27	-	[-/CC]	-	1/176 (0.57)	60.5	1 FL	0/459 (0)
*MAPT*-10	GTC-aTC	149280278	V287I	-	2/176 (1.14)	64.5-70.5	1 SE and 1 FL	0/534 (0)
*MAPT*-4A	TCG-TtG	73314997	S318L	-	1/176 (0.57)	64.5	1 SE	0/139 (0)
*MAPT*-4A	GGG-aGG	76375268	G213R	-	1/176 (0.57)	64.5	1 SE	0/139 (0)
*MAPT*-4A	GTC-GgC	141120474	V224G	-	3/176 (1.7)	58.5-72.5	1 FE and 2 FL	1/139 (0.72)
*MAPT*-4A	CAA-CgA	63750072	Q230R	-	18/176 (10.23)	50.5-83.5	1 AC, 2 FE, 2 FL, and 13 SE	8/139 (5.8)
*MAPT*-4A	GCC-GtC	-	A297V	-	1/176 (0.57)	59.5	1 FE	0/139 (0)
*MAPT*-7	GCC-aCC	143624519	A152T	-	2/176 (1.14)	57.5	1 AC and 1 SE	0/139 (0)
*PSEN1*-5	ATG-AcG	63751106	M139T	Pathogenic	1/176 (0.57)	47.5	1 SE	0/459 (0)
*PSEN1*-6	TTG-TTt	-	L173F	Pathogenic	1/176 (0.57)	50.5	1 SE	0/459 (0)
*PSEN1*-9	GAA-GgA	17125721	E318G	Not pathogenic	2/176 (1.14)	56.5-65.5	2 SE	3/139 (2.2)
*PSEN1*-11	CTG-gTG	63751416	L392V	Pathogenic	1/176 (0.57)	42.5	1 FE	Not available

^a^Alzheimer Disease & Frontotemporal Dementia mutation database [15]. ^b^This column summarizes the status of carriers in Alzheimer's disease cases. AAO, age at onset; AC, autopsy-confirmed Alzheimer's disease; FE, familial early-onset Alzheimer's disease; FL, familial late-onset Alzheimer's disease; *GRN*, progranulin; *MAPT*, microtubule-associated protein tau; *PSEN1*, presenilin 1; SE, sporadic early-onset Alzheimer's disease; SL, sporadic late-onset Alzheimer's disease.

Four missense mutations (p.M139T, p.L173F, p.E318G, and p.L392V) in *PSEN1 *were validated (Table 2). The p.M139T mutation located in exon 5 of *PSEN1 *and the p.L392V mutation in exon 11 have been shown to be pathogenic in previous studies [5,18-24]. The p.M139T carrier, who had a ε3/ε3 genotype, was a sporadic EOAD case with an AAO of 47.5 years, which is close to the mean AAO of disease in three previously reported families (one French family and two independent Spanish families). The p.L392V mutation, which has been identified in five families (one Italian family, one French, one Japanese, and two unknown), was confirmed in a familial EOAD case with the *APOE *ε3/ε4 genotype and AAO of 42.5, which is similar to the mean AAO in these five families [5,18,19,22-24]. We also found a novel G-to-T transition (g.38816G>T) in exon 6 of *PSEN1*, resulting in a previously described pathogenic p.L173F missense mutation [25]. The carrier of the p.L173F mutation in our study, who was an *APOE *ε3/ε3 carrier, had an AAO of 50.5 years, which is close to the mean AAO in the family reported by Kasuga and colleagues [25]. The p.L173F mutation was shown to be associated with presenile dementia and parkinsonism [25]. The p.M139T and p.L173F mutations in *PSEN1 *were not found in any additional cases or the 459 Spanish controls. The p.E318G mutation was a low-frequency polymorphism observed at equal frequencies in patients and unaffected controls in several previous studies [26-28]. The p.E318G frequency in our study was higher in controls (2.16%, 3 out of 139) than in AD cases (1.14%, 2 out of 176), suggesting that p.E318G might not be a risk factor for clinical AD.

**Figure 2:**
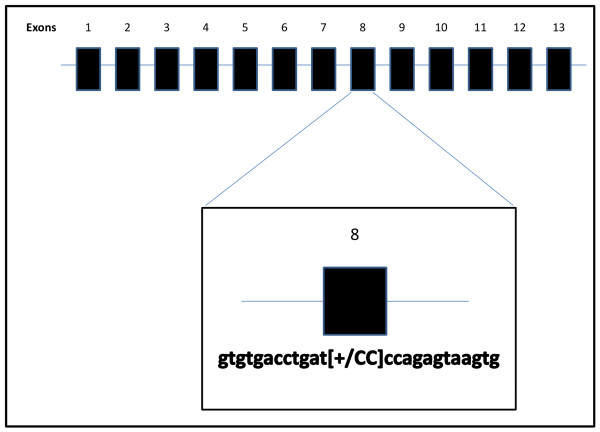
**The transcript of the novel *GRN *mutation**. A splice-site mutation with a CC-insertion (g.10974_10975insCC) in *GRN *exon 8 results in a premature stop codon and nonsense-mediated decay of the resultant mRNA. *GRN*, progranulin.

We also found a novel *GRN *CC insertion, g.10974_10975insCC (Ile256IlefsX27), which was not present in the AD&FTD database, 1000 Genomes Project, SeattleSeq, or EVS (Figure 2). The CC insertion is a frameshift mutation located in exon 8 of *GRN*, introducing a premature stop codon 27 amino acids after the insertion site (Ile256IlefsX27). Considering other frameshift mutations in *GRN *[29-33], we predicted that the mutant allele will be degraded by nonsense-mediated decay, resulting in a loss of one functional allele. To confirm that the novel *GRN *frameshift mutation (Ile256IlefsX27) was related to the diagnosis of AD, we performed segregation analysis, genotyping of additional Spanish controls, and functional analysis. The proband, the carrier of the *GRN *mutation with the *APOE *ε3/ε3 genotype, had an AAO of 60.5 years and was diagnosed with probable AD on the basis of international criteria [12]. One of the proband's siblings, who had a diagnosis of probable AD, is also a carrier of the mutation. Two other non-demented siblings of the proband did not carry the variant. The *GRN *frameshift mutation was absent in 449 older healthy Spanish controls.

**Figure 3:**
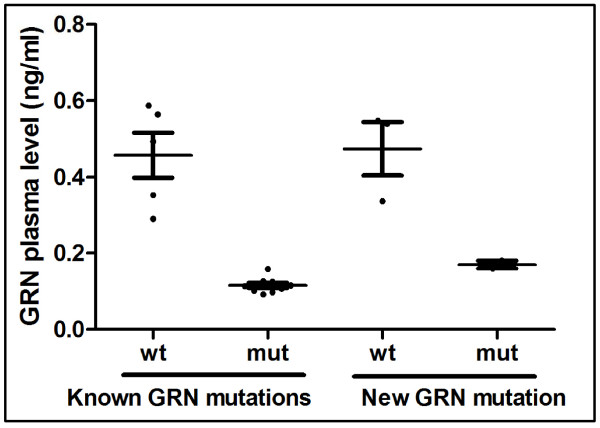
**Enzyme-linked immunosorbent assays comparing *GRN *plasma levels between *GRN *mutation carriers and controls**. Measurement of *GRN *plasma levels for a known *GRN *mutation (left) and the novel *GRN *mutation (right), Ile256IlefsX27, observed in our study compared with Alzheimer's disease cases and non-demented older controls. *GRN*, progranulin; mut, mutant; wt, wild-type.

Previous studies have shown that individuals with pathogenic *GRN *mutations have lower *GRN *plasma levels [30,31,34-38]. Therefore, we tested whether the *GRN *g.10974_10975insCC mutation was associated with low *GRN *plasma levels. The *GRN *plasma levels were measured in 18 individuals, including two non-demented siblings of the proband who did not carry the mutation, 11 carriers of a previously identified *GRN *mutation (as positive controls), and five family members who were non-carriers for *GRN *mutations [35,39,40]. The *GRN *g.10974_10975insCC mutation carrier had an average *GRN *plasma level of 0.125 ng/μL, which is very close to 0.116 ng/μL, the average *GRN *plasma level of a known *GRN *mutation carrier (Figure 3). The two siblings of the carrier of the *GRN *g.10974_10975insCC mutation had an average *GRN *plasma level of 0.463 ng/μL, which is close to the mean *GRN *plasma level for the five family members who were non-carriers of the *GRN *mutation (0.504 ng/μL). Together, these results confirm that the *GRN *g.10974_10975insCC mutation is pathogenic.

Seven missense variants in *MAPT *were further validated in our study: five in exon 4A (p.S318L, p.G213R, p.V224G, p.Q230R, and p.A297V), one in exon 7 (p.A152T), and one in exon 10 (p.V287I). The frequency of each variant in cases and controls is listed in Table 2. p.G213R, p.V224G, p.Q230R, and p.S318L reside in exon 4A of *MAPT *and are present in SeattleSeq, EVS, or 1000 Genomes Project, suggesting that they do not affect risk for AD. The p.A297V mutation also located in exon 4A was not found in any annotation tool, and thus the functional role of p.A297V remains unclear. The p.A152T variant located in exon 7 was suggested in a recent study to be a risk factor for FTD-spectrum and AD in a total of 15,369 subjects [41]. Another recent study shows that the p.A152T mutation increases risk for developing neurodegenerative diseases by influencing tau accumulation [42]. We found two p.A152T carriers among the cases (one with ε3/ε3 and one with ε4/ε4 genotype) and none in the controls. Segregation analysis showed that the healthy 69-year-old sibling of the AD proband did not carry the p.A152T mutation. No family history for the other MAPT p.A152T carrier was reported, but autopsy was available for this individual. Fresh brain weight was 924 g. A macroscopic examination revealed moderate global cortical atrophy, and serial coronal sections of the left hemisphere showed moderate ventricular dilatation and atrophy of the medial temporal lobe, particularly in its anterior segment. A histological examination based on immunohistochemistry for tau and beta-amyloid revealed a high density of neuritic plaques in associative isocortical areas, a dense distribution of neurofibrillary tangles consistent with Braak stage VI, and moderate amyloid angiopathy. Additionally, immunostaining for alpha-synuclein displayed abundant Lewy bodies and neurites at the amygdaloid complex and enthorrinal cortex and a moderate density of inclusions in the parahippocampal and cyngular cortices and in brainstem nuclei. These findings are consistent with a neuropathological diagnosis of Alzheimer's type changes with a high probability of disease, combined with Lewy type pathology of the limbic predominant subtype [43].

These results suggest that p.A152T contributes to an increased risk associated with clinical AD. The p.S318L variant, located in exon 10 of *MAPT*, was found in two cases (one with ε3/ε3 and one with ε3/ε4 genotype) and none of 534 ethnicity-matched controls, suggesting that p.S318L might increase the risk for clinical AD. However, studies with a larger sample size are needed to confirm its role in AD risk.

## Discussion

Our study has shown that pooled-DNA sequencing can effectively identify known and novel pathogenic variants. So far, around 344 different mutations have been identified in *APP*, *PSEN1*, *PSEN2*, *GRN*, and *MAPT *according to the AD&FTD mutation database. In this study, we identified four pathogenic variants (p.M139T, p.L173F, and p.L392V in *PSEN1 *and g.10974_10975insCC in *GRN*), two of which were novel (p.L173F in *PSEN1 *and g.10974_10975insCC in *GRN*). These four pathogenic variants explain 2.33% of our clinical AD cases. However, the lack of additional familial cases prevents us from performing segregation analysis for other variants (*MAPT *p.V287I and p.A152T), and therefore their effect on risk for disease is unclear. Additional genetic and functional analyses are required to confirm the effect of these variants on risk for AD.

On the other hand, for the *GRN *g.10974_10975insCC insertion, we confirmed that this novel mutation segregates with disease status. This rare insertion mutation was not found in 459 older healthy Spanish controls. In addition, our results from functional analyses were concordant with the known biological function of other known pathogenic *GRN *variants. The *GRN *frameshift mutation creates a premature stop codon that results in nonsense-mediated decay leading to a decrease in *GRN *mRNA levels and protein levels in plasma and cerebrospinal fluid [7,29-33]. We found that the *GRN *g.10974_10975insCC mutation carriers had extremely low plasma *GRN *levels, confirming the functional role of this novel mutation.

In *MAPT*, we found the p.A152T variant in exon 7 in two cases and no controls. Recently, this variant was reported to be a risk factor for FTD and AD [41]. The p.A152T variant was found in 0.69% of AD cases and 0.3% of controls (odds ratio = 2.3, *P *value = 0.004) [41]. In our segregation analysis, p.A152T was absent in the healthy 69-year-old sibling of the proband, suggesting that p.A152T does segregate with disease in this family. The p.V287I mutation was found in one chromosome out of 10,753 (corresponding to a frequency of 0.0186%) in the EVS database. We found this variant in two cases (1.14%), which is a much higher frequency than that observed in the EVS database, suggesting that this variant could be associated with increased risk for disease. So far, all of the *MAPT *variants in exon 4A were reported to be non-pathogenic (six out of six) in the AD&FTD database [44-47]. In addition, previous studies have shown that the *MAPT *exon 4A is not expressed in the adult human cerebral cortex [48,49]. Although all six of the *MAPT *variants in exon 4A found in this study (including p.S318L, p.G213R, p.V224G, p.Q230R, and p.A297V) have higher frequencies in cases than in controls, these variants are more likely to be non-pathogenic according to previous findings and records.

## Conclusions

In summary, we have identified two novel pathogenic variants: one in *PSEN1 *gene and the other in *GRN*. We also conducted functional studies to validate the pathogenicity of the *GRN *variant. Our findings are consistent with those that were previously reported by our group in a different population [7] and that concluded the following: (a) there are likely more novel pathogenic variants causing an AD phenotype to be discovered in the AD (*APP*, *PSEN1*, and *PSEN2*) and FTD (*MAPT *and *GRN*) genes, (b) pathogenic variants located in such 'neurodegeneration' genes could be responsible for both EOAD and LOAD, and (c) mutations in *GRN *and *MAPT*, which have been considered traditionally 'FTD genes', can be present in individuals with a clinical presentation indistinguishable from that of typical AD. Pathological information for the individual carriers for the novel *GRN *insertion and the novel *MAPT *variants was not available, so the AD diagnosis was based solely on clinical assessment. Given other reports, it is more likely that these individuals are amnestic FTD cases but had a clinical presentation indistinguishable from that of AD [7]. Identification of mutations in *MAPT *and *GRN *can help to make a more accurate diagnosis in these individuals. These results highlight the necessity of screening both AD and FTD genes when autopsy confirmation of diagnosis is unavailable in demented individuals.

## Abbreviations

AAO: age at onset; AD: Alzheimer's disease; AD&FTD: Alzheimer Disease & Frontotemporal Dementia; *APP*: amyloid-β precursor protein; bp: base pairs; ELISA: enzyme-linked immunosorbent assay; EOAD: early-onset Alzheimer's disease; EVS: Exome Variant Server; FTD: frontotemporal dementia; *GRN*: progranulin; LOAD: late-onset Alzheimer's disease; *MAPT*: microtubule-associated protein tau; NGS: next-generation sequencing; PCR: polymerase chain reaction; *PSEN1*: presenilin 1; *PSEN2*: presenilin 2; SPLINTER: short indel prediction by large deviation inference and nonlinear true frequency estimation by recursion.

## Competing interests

The authors declare that they have no competing interests.

## Authors' contributions

CC helped to conceive and design the experiments, to provide human samples and reagents, to analyze data, and to draft the manuscript. PP helped to conceive and design the experiments, to provide human samples and reagents, and to revise the manuscript. SCJ helped to conceive and design the experiments, to perform experiments, to acquire and analyze data, and to draft the manuscript. The Ibero-American Alzheimer Disease Genetics Group helped to provide human samples and reagents. SC helped to provide human samples and reagents, to perform experiments, and to acquire data. AG helped to provide human samples and reagents and to revise the manuscript. BC helped to perform experiments, to acquire data, and to draft the manuscript. BAB helped to analyze data and to revise the manuscript. All authors read and approved the final manuscript.
